# Factors associated with use and non-use of mosquito nets owned in Oromia and Amhara Regional States, Ethiopia

**DOI:** 10.1186/1475-2875-8-264

**Published:** 2009-11-23

**Authors:** Carol A Baume, Richard Reithinger, Sara Woldehanna

**Affiliations:** 1 AED, 1825 Connecticut Ave NW Washington, DC 20009, USA; 2 President's Malaria Initiative, U.S. Agency for International Development, Addis Ababa, Ethiopia; 3 2501 McComas Ave, Kensington, MD 20895, USA

## Abstract

**Background:**

Many countries across sub-Saharan Africa are rapidly increasing insecticide-treated net (ITN) coverage to combat malaria, but systematic data on the use of those ITNs and the factors affecting this use are scarce.

**Methods:**

A household survey was conducted during malaria season in 23 communities of Amhara and Oromia Regional States, Ethiopia, stratified by degree of urbanization (rural, peri-urban, or urban), whether or not they received indoor residual spraying (IRS), and whether or not free nets had been distributed. Descriptive statistics as well as univariate and multivariate logistic regression analyses were used to describe household net ownership and identify factors associated with use or non-use of nets already in the household. A qualitative component consisting of observations of ITNs in households and several open-ended questions provided further understanding of the reasons for ITN use and non-use.

**Results:**

Of 857 surveyed households, 91% owned at least one ITN, but only 65% of ITNs owned had been used the prior night. The multivariate analysis found that the factors significantly associated with an ITN being used were regional state (Amhara) (Odds Ratio [OR] = 0.61; 95% Confidence Interval [C.I.] 0.43 - 0.86]; p < 0.01), residing in a house sprayed with IRS (OR = 1.89; 95% C.I. 1.36 - 2.63; p < 0.001), age of ITN (<12 months) (OR = 0.55; 95% C.I. 0.37 - 0.82; p < 0.01), shape (conical) (OR = 2.27; 95% C.I. 1.10 - 4.68; p < 0.05), and paying for the ITN rather than receiving it free (OR = 2.16; 95% C.I. 1.32 - 3.53; p < 0.01). The most common reasons for ITN non-use identified through the qualitative component of the study were: there are few mosquitoes around or malaria is not a serious problem; the ITN is no longer effective; ITN is in poor condition; the ITN is being saved. Observations showed many ITNs hanging incorrectly, and some being used for purposes other than as a bed net.

**Conclusion:**

The very high ITN ownership in the study areas suggests that a strategy targeting free nets to rural and poor households combined with support for the commercial sector is an effective means of achieving high coverage. The data suggests that use of ITNs owned could be increased by distribution of conical ITNs, continued development of the commercial sector, replacement schemes for worn-out ITNs, assistance with hanging of ITNs, and communication addressing misperceptions about ITNs.

## Background

Insecticide-treated mosquito nets (ITNs) have proven to be highly effective in preventing malaria. Consistent use of ITNs can reduce malaria transmission by up to 90% and avert as much as 44% of all-cause mortality among children under five years of age [1,2]. Use of ITNs among pregnant women is associated with lower prevalence of malaria infection, fewer premature births and significant reductions in all-cause maternal anaemia [3-6]. ITNs used to require periodic re-treatment to maintain effectiveness, but in recent years a type of ITN, the long-lasting insecticidal net (LLIN), has been developed that - in experimental settings - lasted 20 washings or 3-4 years [7].

Mosquito nets of any kind are relatively new to Ethiopia. In 2000, the Demographic and Health Survey (DHS) found that only 1.1% of households nationwide owned a net [8]; in the 2005 DHS, the percentage was 5.7% [9]. Concerted interventions to promote ITNs began in 2004 and focused on making affordable ITNs available through the commercial sector, both at commercial prices and subsidized prices via direct subsidy or vouchers to vulnerable groups. Since 2005, there has been a large infusion of donor money - primarily from the Global Fund to Fight AIDS, Tuberculosis and Malaria (GFATM) - to the Government of Ethiopia's Federal Ministry of Health (FMOH) for dramatically expanding malaria control interventions in the country. This included the free distribution of approximately 20 million LLINs to households in primarily rural malaria-endemic areas, with an aim for an average of two ITNs per household.

Although ITN distribution has been massively expanded since 2005, there is little information on use of nets owned and reasons for non-use, either in Ethiopia or other countries. Most studies of ITN use attempt to explain why vulnerable groups (i.e. children under five or pregnant women) are or are not under an ITN [10-15], or describe which household members use the household's ITN(s) [16]. Those studies vary widely in terms of study protocol, and type and number of potential explanatory variables assessed, and there are no common conclusions except that ITN use is higher in the rainy season or when mosquito density is high. The one study encountered in the literature that investigated the proportion of ITNs used the prior night was an early intervention trial in Kenya where all households had received ITNs along with instruction in hanging, provision of hanging supplies (e.g. twine, nails), and education [17]. That study found that 30% of ITNs distributed were not used the prior night, and that heat was the main reason given for non-use. As far as could be ascertained, no other study has been reported in the literature that used ITNs as the unit of analysis. No study was found that took into account ITN characteristics as possible explanatory variables for use.

The objective of the present study was to investigate ITN use and factors related to use of ITNs owned. Understanding why ITNs already in the household are not used is essential for refining ITN distribution programmes and for developing effective information, education, communication/behaviour change communication (IEC/BCC) activities to maximize the impact of ITNs in reducing malaria morbidity and mortality.

## Methods

### Study setting

Approximately 75% of Ethiopia's landmass is malarious, with malaria primarily associated with altitude (below 2500 meters) and rainfall [18-21]. Transmission tends to occur twice a year, after the short and long rainy seasons in March-May and July-September, respectively, but epidemics occur roughly every 5-8 years. In 2006, malaria accounted for approximately 29% of in-patient deaths, and represented a significant economic burden on affected populations [22].

### Study approach

The study used both quantitative and qualitative methods. The quantitative component was a household survey that included questions on household demographic characteristics, whether or not a house had received indoor residual spraying (IRS), malaria knowledge, ITN ownership, and questions regarding the characteristics and use of each household ITN. A qualitative component was integrated into the survey instrument and included observation of nets in the home as well as open-ended questions related to use. The open-ended questions were included to provide in-depth information on use and non-use of ITNs and allow for exploration of issues that might arise outside of the quantitative survey questions. When a household had an ITN that had not been slept under the prior night, the interviewer asked why, and was allowed to probe for clarification and ask follow-up questions. Interviewers also observed if and how ITNs were hanging, and what their condition was.

The field team consisted of six interviewers from the Addis Ababa University. The team was kept small and travelled together as a unit for quality control purposes. In preparation for fieldwork, the Principal Investigator (PI) discussed the rationale for the study and provided background information on malaria and ITNs. Using a draft instrument, the team spent four days practicing administering the instrument and testing and revising it. Emerging findings were discussed at the end of each day, and a summary of findings written. The PI remained with the team during the first four days of actual data collection to continue refining the skills of the team and discuss and compare results among communities. The team continued collecting data on its own for another four weeks. The PI returned at the end of fieldwork to work with two team members to re-visit some communities for data verification and to write up the qualitative results.

### Sample

The study took place in communities below 2500 meters in Amhara and Oromia Regional States, Ethiopia, during October 2007, after the rainy season when mosquito density and malaria transmission is high. The main urban areas in Amhara and Oromia were included in the study, with the exception of Addis Ababa (the capital), and Dessie town, both of which are located at altitudes too high for malaria transmission. Since the purpose of the study was to look at ITN use under different conditions, districts and communities were listed and stratified according to: (i) whether they were urban, peri-urban or rural; (ii) whether or not they had been targeted for IRS; and (iii) whether or not the community had received free ITNs. 'Urban' areas were those having a population exceeding 2,500, a post office and access to public services including telephones; 'peri-urban' areas were those with a population exceeding 2,500, but lacking a post office and public services; 'rural' areas were those with at least 75% of the households engaged in agriculture. There were few areas where IRS had been implemented, so all of those areas were included in the sample. For logistical reasons, communities with known extreme difficulty of access were excluded. Purely pastoral areas were also excluded, because their distinct behavioural patterns are likely to affect bed net use, and because their relatively small population size would not make results applicable for planning region-wide IEC/BCC campaigns. Otherwise, communities were randomly selected within strata. In order to assure a sufficient number of cases (i.e. households with ITNs) within strata, 30 households were randomly selected per community. Upon arrival in a selected community, the interviewers dispersed in different directions. In densely settled areas, every fourth house was selected and in sparsely settled areas, every other household was selected until 30 households per community were interviewed. The respondent could be either the husband or wife, but was most often (80% of the time) the wife or female head of household. Verbal consent was obtained of study participants. It should be noted that the sampling strategy was designed to understand ITN use in different contexts, not necessarily to yield accurate point estimates of ownership or even of overall level of ITN use within Amhara and Oromia Regional States.

### Data management and analysis

Survey questionnaires were brought back to Addis Ababa for data entry. The data were analyzed in SPSS 16.0 (SPSS, Inc., Chicago, IL) and STATA 9.0 (Stata Corporation, College Station, TX). The data were entered with each household/respondent as a case (i.e., household-level file). This file was used for basic description of the sample, including levels of ITN ownership. Differences between sample proportions were analysed using the chi-square test. For the analysis of ITN use, the data were restructured into a separate file with each net/ITN owned as the unit of analysis (i.e., net-level file). For households where all household members slept under an ITN and additional ITNs were owned, the "extra" ITNs were taken out of the analysis since there was no one to use them. Whether or not the ITN was used the prior night was the dependent variable. The association of each possible explanatory variable with the dependent variable (ITN used or not the prior night) was tested by estimating univariate odds ratios (OR).

The explanatory variables fell into the following categories:

**(1) Household background characteristics**: regional state (Amhara or Oromia); urban, peri-urban, or rural location; whether or not a household received IRS in the past year; presence of a child under five; presence of a pregnant woman; whether respondent said malaria occurred in household within the prior year; household ITN density (number of ITNs owned divided by number of household members); whether or not the household bought an ITN, obtained an ITN with a voucher, or received one for free in the past four years.

**(2) Respondent knowledge and beliefs about malaria and ITNs**: knowledge that mosquitoes cause malaria; knowledge of fever/chills as a key sign of malaria; perceptions of the safety of ITNs for young children and pregnant women.

**(3) Characteristics of the ITN**: condition in terms of wear; age; size; shape; colour; free, purchased with voucher, or purchased without voucher.

A multivariate model was developed using generalized estimating equations (GEE), a method for estimating regression parameters when data are potentially correlated and when the outcome variable is not continuous or normally distributed. GEE was used for this analysis because of the possible clustering of ITN use patterns within households. In addition, in GEE a logit link function with an underlying binomial distribution could be specified to model - in this case, the dichotomous outcome variable in this study. An initial "base" GEE model of ITNs clustered within households utilizing robust covariance estimator was constructed, which included all independent variables found to be significant in the univariate analysis. Variables not significant at p < 0.05 level were then eliminated one by one in order of least significance. Afterward, potential explanatory variables that were not found to be significant in the univariate models were evaluated one by one for inclusion into the model. Finally, the parameter correlation matrix of the resulting model was evaluated to assess multi-colinearity and to determine which if any of the variables should be eliminated from the model. The odds ratios (the odds of an ITN being used the prior night under various conditions defined by the independent variables) and 95% confidence intervals for the mean response were computed from the final GEE model.

The qualitative analysis was conducted in several ways. During fieldwork, the field team discussed findings at the end of the day, and wrote summaries of findings after completing a study area. After the fieldwork, the responses to open-ended questions that were written on the questionnaires were entered as text into the data file along with the quantitative data. Each response was also coded; a response could be given more than one code if more than one reason for not using the ITN was given. Both the actual text and the codes were stratified by regional state as well as by urban, peri-urban, and rural category to look for differences and trends by groups.

## Results

### Characteristics of the sample

A total of 857 households were included in the study: 531 in Oromia and 326 in Amhara. Household size ranged from 1-15, with a mean of 5.27 people (95% C.I. 5.11 - 5.42). Of the 4516 people in the surveyed households, 648 (14%) were children under five and 70 (2%) were pregnant women.

### ITN ownership

Of surveyed households, 91% owned at least one ITN (Table 1). ITN ownership was higher in Amhara (96%) than Oromia (88%) (Yates-corrected chi-square test; OR = 3.05 [95% C.I. 1.63-5.81]; p < 0.001). The more urbanized the area (i.e. urban vs. peri-urban vs. rural), the less likely the household was to own an ITN (chi-square test for trend, p < 0.001).

**Table 1:** Percent of households owning at least one ITN

	Number of HH owning 1 or more ITNs (%)	Number of HH owning 1 or more free ITNs (%)	Number of HH owning 1 or more purchased ITNs (%)	N Households
**TOTAL**	**779 (91)**	**639 (75)**	**200 (23)**	**857**

				

**AMHARA**	**312 (96)**	**246 (76)**	**96 (29)**	**326**

**Urban**	57 (89)	5 (8)	54 (84)	64

**Peri-urban**	57 (97)	52 (88)	21 (36)	59

**Rural**	198 (98)	189 (93)	21 (10)	203

				

**OROMIA**	**467 (88)**	**393 (74)**	**104 (20)**	**531**

**Urban**	88 (69)	26 (20)	67 (53)	127

**Peri-urban**	78 (86)	76 (84)	16 (18)	91

**Rural**	301 (96)	291 (93)	21 (7)	313

				

**N (households)**	857	857	857	

Of ITN-owning households, 63% owned more than one ITN. The mean number of ITNs owned by ITN-owning households in Amhara was 1.85 (95% CI 1.78 - 1.91) and in Oromia 1.78 (95% CI 1.72 - 1.83). In both regional states, households owning more than one ITN were most likely to be found in rural or peri-urban areas (64%) rather than in urban areas (37%) (Yates-corrected chi-square test; OR = 3.01 [95% C.I. 2.13-4.26]; p < 0.001).

The total number of ITNs owned by households in the sample was reported to be 1,405 and 95% of those were seen by the interviewers, and 84% were LLINs. ITNs were classified as free or purchased; the latter included ITNs sold at subsidized prices, those bought with vouchers, and those bought at full commercial prices. Overall, 75% of households owned a free ITN and 23% owned a purchased ITN. Purchased ITNs were more common in Amhara (29% of households) than in Oromia (20%) (Yates-corrected chi-square test; OR = 1.71 [95% C.I. 1.23 - 2.39]; p < 0.01). Compared to households in rural areas, households in peri-urban or urban areas were more likely to have purchased an ITN in the last four years (chi-square test for trend, p < 0.001). Since most ITNs owned were ITNs distributed free to beneficiaries by the FMOH, the vast majority of ITNs were blue rectangular LLINs (PermaNet 2.0 ^®^, Vestergaard Frandsen, Denmark) distributed within the prior two years. The average age of ITNs owned was 1.74 years (95% C.I. 1.64 - 1.83).

Of surveyed households, 9% did not own any ITNs. In open-ended discussions with interviewers, no single dominating reason was given for not having an ITN. Some respondents stated that that they did not need an ITN because malaria was not a problem for their family or in their community; others were unaware of free ITN distribution or were unable to collect ITNs during the distribution period; others said they were waiting to be given a free ITN or they were unable to afford an ITN.

### ITN use

Of the 1,405 ITNs owned by surveyed households, 1,391 (99%) had complete data associated with use and were included in the analyses described below. Of ITNs owned, 65% reportedly had someone sleeping under them the prior night (Table 2). Some ITNs in surveyed households had never been used: 11% in Amhara and 20% in Oromia (Yates-corrected chi-square test; OR = 0.58 [95% C.I. 0.41-0.82]; p < 0.001).

**Table 2:** ITNs used, among ITNs currently in the household

	Number of all ITNs used last night (%)	Number of free ITNs used last night (%)	Number of purchased ITNs used last night (%)	Number of ITNs never used (%)	N ITNs
**TOTAL**	**871 (65)**	**669 (63)**	**176 (76)**	**220 (16)**	**1342**

					

**AMHARA**	**395 (73)**	**283 (71)**	**100 (79)**	**61 (11)**	**545**

**Urban**	87 (81)	5 (50)	77 (85)	2 (2)	107

**Peri-urban**	75 (73)	62(78)	9 (50)	19 (18)	103

**Rural**	233 (70)	216 (70)	14 (82)	40 (12)	335

					

**OROMIA**	**476 (60)**	**386 (59)**	**76 (72)**	**159 (20)**	**797**

**Urban**	90 (63)	24 (53)	55 (71)	22 (15)	143

**Peri-urban**	70 (50)	59 (48)	8 (73)	34 (25)	139

**Rural**	316 (61)	303 (62)	13 (72)	103 (20)	515

					

**IRS HH**	**306 (70)**	**286 (70)**	**19 (91)**	**60 (13)**	**458**

**NON-IRS HH**	**565 (62)**	**383 (59)**	**157 (74)**	**164 (18)**	**871**

					

**N (ITNs)**	1342	1061	232	1342	

### Univariate analysis

Results of the univariate analyses of ITN use, clustered at the household level, are presented in Table 3. Of the household variables explored, regional state, number of household ITNs, IRS status, whether household received a free ITN, and whether household had bought an ITN were significantly associated with ITN use the prior night. Of respondent variables, knowledge that mosquitoes transmit malaria was significantly associated with ITN use but knowing signs of malaria was not. Of ITN characteristics, the variables significantly associated with use were ITN age, condition, shape, colour, and whether the ITN had been purchased (with or without voucher) or had been given to a household for free. Whether an ITN was free or purchased was correlated with ITN colour (Pearson's r = 0.70; p < 0.01), shape (Pearson's r = 0.59; p < 0.01) and where it was obtained (Pearson's r = 0.79; p < 0.01).

**Table 3:** Univariate analysis: estimates of the effect of each explanatory variable on the probability that a net was used the night prior to the survey

		Total	%	OR	P	95% CI
**Household Characteristics**						

Regional State***	Amhara	570	40.98			
	Oromia	821	59.02	0.61	<0.001	0.47 - 0.80

Locale	Rural	876	62.98			
	Peri-urban	261	18.76	0.74	0.081	0.53 - 1.04
	Urban	254	18.26	1.37	0.100	0.94 - 1.99

Household number of children <5	0	610	43.85			
	1	781	56.15	1.15	0.308	0.88 - 1.49

Household number of pregnant women	0	1286	92.45			
	1	105	7.55	1.05	0.828	0.64 - 1.74

Household size - number of persons	1-4	458	32.93			
	5-7	649	46.66	0.73	0.609	0.21 - 2.46
	>7	284	20.42	0.48	0.198	0.15 - 1.47

Number of household nets*	1	289	20.78			
	2	766	55.07	0.71	0.025	0.53 - 0.96
	>3	336	24.16	0.80	0.251	0.54 - 1.17

Net density: #nets/#people in HH	0.00 - 0.25	316	22.72			
	0.26 - 0.50	855	61.47	1.17	0.317	0.86 - 1.59
	>0.51	220	15.82	0.77	0.214	0.51 - 1.16

Household member had malaria in prior year	No	771	55.43			
	Yes	600	43.13	1.24	0.114	0.95 - 1.16

Household received IRS in the past year**	No	948	68.15			
	Yes	443	31.85	1.47	0.007	1.11 - 1.94

Household received free ITNs**	No	191	13.73			
	Yes	1190	85.55	0.55	0.005	0.36 - 0.83
	No	1395	97.42			

**Household got ITN with a voucher**	Yes	37	2.37	2.11	0.051	1.00 - 4.47
Households bought ITNs**	No	1084	75.70			
	Yes	348	24.30	1.52	0.01	1.11 - 2.08

**Respondent's Knowledge**						

Knows mosquitoes transmit malaria*	No	295	21.21			
	Yes	1084	67.86	1.41	0.029	1.04 - 1.91

Knows signs of malaria	0	41	2.95			
	1-2	994	71.46	1.55	0.184	0.81 - 2.97
	3+	346	24.87	1.84	0.083	0.92 - 3.66

**ITN Characteristics**						

Net age (months)*	0 - 6	445	31.99			
	6 - 12	124	8.91	1.92	0.015	1.13-3.27
	>12	723	51.98	1.01	0.968	0.76-1.33

Net condition***	New or like new	593	42.63			
	Worn but no holes; patched	498	35.80	0.89	0.433	0.66 - 1.20
	Small holes	92	6.61	1.10	0.717	0.65 - 1.87
	Large holes	42	3.02	0.33	0.001	0.17 - 0.65

Net size	Single/cot	13	0.93			
	Double	931	66.93	0.90	0.865	0.26 - 3.07
	Triple/family size	339	24.37	0.68	0.542	0.20 - 2.35

Net shape***	Rectangular	1177	84.62			
	Conical	126	9.06	2.62	<0.001	1.55 - 4.42

Net colour*	Blue	1084	77.93			
	Green	87	6.25	0.68	0.137	0.41 - 1.31
	White	111	7.98	1.80	0.022	1.09 - 2.98

Net was purchased***	No	1096	78.79			
	Yes	237	17.04	1.84	0.001	1.28 - 2.65

Significance: * p <= 0.05; ** p <= 0.01; *** p <= 0.001

### Multivariate analysis

The final GEE model showed that five of the explanatory factors considered had a significant association with an ITN being used the prior night (Table 4). Regional state remained significant: ITN use was lower in Oromia than in Amhara (OR = 0.61 [95% C.I. 0.43-0.86]; p < 0.01). In addition, an ITN in a household that had been sprayed in the last year was more likely to be used than one from a household that had not received IRS (OR = 1.89 [95% C.I. 1.36-2.63]; p < 0.001). The age of the ITN was associated with use; ITNs obtained more than 12 months ago were less likely to be used than those obtained less than six months ago (OR = 0.55 [95% C.I. 0.37-0.82]; p = 0.01). Conical ITNs were more likely to be used the prior night compared to rectangular ITNs (OR = 2.27 [95% C.I. 1.10-4.68]; p < 0.05), and a purchased ITN was more likely to be used than one obtained free (OR = 2.16 [95% C.I. 1.32-3.53]; p < 0.01). Although colour of ITN was found to be significant in the multivariate analysis, it was removed from the final model because of multi-collinarity in model estimates; colour was highly correlated (r > 0.5) with both free-bought status and shape, since all ITNs distributed free in the prior two years were blue rectangular ITNs.

**Table 4:** Multivariate analysis: estimates of the effect of explanatory variables on the probability that a net was used or not used in the night prior to the survey

	OR (95% C.I.)	P
Region (compared to Amhara)**		

*Oromia*	0.61 (0.43- 0.86)	0.005

		

HH sprayed with IRS (compared to No)***		

*Yes*	1.89 (1.36 - 2.63)	0.000

		

ITN age (compared to 0-6 months)**		

*6 - 12*	0.81 (0.56 - 1.18)	0.272

*>12*	0.55 (0.37 - 0.82)	0.003

		

ITN shape (compared to rectangular)*		

*Conical*	2.27 (1.10 - 4.68)	0.027

		

ITN Purchase status (*compared to Free*)**		

Purchased	2.16 (1.32 - 3.53)	0.002

Significance: * p <= 0.05; ** p < = 0.01; *** p < = 0.001

### Qualitative results

The primary reasons for not using an ITN owned emerging from observations and responses to open-ended questions were the following:

**(1) The perception that the ITN had lost its effectiveness or needed re-treatment**: This was the single most frequent reason for not using an ITN owned, but was more commonly given in rural than other areas. The perception that the ITN was no longer effective had to do with not seeing dead insects, or from the idea that the ITN needed re-treatment. Respondents explained: *"It is expired; it needs to be re-treated." "It is not killing insects currently so we are not using it now." "The chemical is no longer active." "Previously we were using the net when the treatment was effective, but at this time the treatment is not effective so we are not using it." "The net should be retreated, but we don't have the chemical so we are using the net as a bed sheet." "I used it before but the chemical expires after six months." "I washed the net and think it is no longer effective to kill mosquitoes."*

**(2) The perception that malaria or mosquitoes were not a serious problem**: This perception tended to cluster by community; that is, it was either very common or very uncommon to hear it expressed in a given community. It was especially common to hear in urban Oromia and was reflected in statements such as *"Malaria used to be rampant, but not anymore", "No one in this household has gotten malaria in the past two years" *or *"There are not many mosquitoes nowadays."*

**(3) Nets in poor condition**: Especially in rural areas, many nets, even those acquired within the prior year, were observed to have holes and tears and to be dirty. Respondents commented about the condition of their ITN, saying it was in too poor a condition to use. *"The nets are old and have holes and do not prevent mosquitoes." "The net is completely worn out." "The net is torn and out of function." *Some ITNs were thought to be too dirty to hang and had been taken down for washing but had not yet been washed, or had been washed but not re-hung.

**(4) Difficulty of hanging ITNs in traditional houses**: Observation showed that several characteristics of local houses make hanging an ITN difficult or unfeasible. In the round houses with a conical roof and a high point in the center, it is especially difficult to find four hanging points that rectangular-shaped ITNs require. *"It is difficult to hang in the place where our children sleep. I have been told by my husband to use it if we make a bed for them. If it had been the conical one it would have been convenient to hang." "It is quite difficult to hang." *The interior living space is usually small and used for multiple purposes, and respondents reported that it was not practical to put up and take down an ITN daily. Children usually sleep on mats on the floor that are rolled up during the day to make room for other activities including cooking. In rural areas, sheep and goats are also reportedly brought in the house at night, taking up further space. *"The room is not adequate to stretch the nets." "It is inconvenient to use in my house because the house is crowded and there is smoke which may make the net get dirty." "The net is unhung because I fear the smoke will damage the net." "The room is used as a kitchen so the net may get worn out and get dirty." *Even if there is space for one ITN, there is seldom space for more than one. *"Since there is no space for the second net, we keep it in the pack." "The room is not sufficient to use two nets." *In addition, when ITNs are hung, most hanging ITNs observed were not hung in a way that would provide good protection for the users. Often the ITN does not reach the sleeping place, especially if the sleeping place is a mat on the floor rather than a raised bed. The gap can be as much as two to three feet. *"The net is short and difficult to hang." "The net can't hang over where the children sleep."*

**(5) Misinformation and lack of information**: There was much confusion as to whether ITNs could be washed and whether they had to be re-treated. In rural Oromia, especially, people said they were told that ITNs needed to be re-treated after six months.

**(6) Saving ITNs for the future**: Respondents explained they were leaving some ITNs unopened to save them for the future, some citing uncertainty of receiving replacement ITNs, or because ITNs can be exchanged for cash. *"The other net is still in the pack; I want to use one at a time." "It [the ITN] is in the pack for the future." "I want to use one after the other." "I will use it when I am pregnant." *Most just said the ITN was still in the package and could not explain why. *"We keep it unopened." "We keep both nets in the package."*

**(7) ITNs being used for other purposes**: ITNs in some communities were observed being used as ceiling covers, bed covers, room dividers, curtains, door curtains, or tablecloths. Some people used the ITNs to dry grain, or tore the ITNs into strips for tying cattle to a tree. Using ITNs for other purposes was not a common problem overall, but it was concentrated in a minority of communities.

## Discussion

In line with the Global Malaria Strategy and with the substantial increase in funding support for malaria prevention and control programmes, countries across sub-Saharan Africa are dramatically increasing their ITN coverage. However, ITN ownership will have little impact on the burden of malaria unless people sleep under them and many large-scale programmes have encountered challenges in ITN acceptance, and consistent use [23]. Although an increasing number of studies have documented ITN ownership [24], including in Ethiopia [25,26], few studies have systematically investigated ITN use.

Clearly, ITN ownership in surveyed study areas in Amhara and Oromia is high. This reflects the massive scale-up efforts in ITN distribution in the past four years, primarily implemented by the FMOH and other in-country stakeholders, including the United Nations Children's Fund, the Carter Center, Population Services International and AED NetMark. Even though the FMOH distribution scheme focused on rural areas, urban areas showed high rates of ITN ownership as well, with households purchasing ITNs at subsidized or full commercial prices. This suggests that a segmentation strategy targeting free ITNs to rural and poorer households combined with support for the commercial sector in urban and better-off areas optimizes ownership. Although ITN ownership was high, it was lower in Oromia than in Amhara. Amhara had a higher percent of households owning at least one net, had more nets owned per net-owning household, and used a higher percentage of nets owned than Oromia, probably because of longer and more intensive history of ITN promotion in Amhara than in Oromia, suggesting that the length and intensity of promotion has "paid off."

Of ITNs enumerated, 35% owned had not been used the prior night, including 16% that had never been used, even though the survey was conducted following the peak transmission season when use is likely to be highest. The quantitative analyses showed that, in addition to regional differences, whether the household had been sprayed with IRS, and characteristics of the ITN - age, shape, and free *vs*. purchased - had a significant impact on whether an ITN net was used. Although some of these variables and their association with ITN use is intuitive, data showing a statistically significant association of these variables with ITN use do not exist in the literature. Further, the qualitative results provide explanations of these associations. Thus, for example, the finding that ITNs less than six months old were more likely to be used than those over a year old is logical in that newer ITNs are likely to be in better condition and have more visible mosquito-repelling power than older ITNs. A common reason given for not using the ITN was that it was '*no longer effective'*. It is possible that an ITN's diminishing knock-down effect over time was perceived as a sign of ineffectiveness, or possibly prior messages about the importance of re-treating nets have buttressed the idea that effectiveness of nets is temporary. Whether the perceived loss in effectiveness is in fact true is unknown, but studies in other settings have shown that loss of insecticide can occur over time following regular handling or exposure to environmental factors such as dirt and soot [27]. Given the widespread comments by respondents that their ITNs were not effective, it may be useful to conduct bioassays of ITNs in rural areas after two years of use, and adjust the frequency of ITN distribution or disseminate messages concerning washing if necessary.

Similarly, field observations help explain the finding that conical ITNs were much more likely to be used than rectangular ITNs. Conical ITNs use a single hanging point for hanging, whereas rectangular ITNs require four hanging points and sometimes extra rope for reaching an attachment point. It is often difficult to find attachment points in rural houses, as nails easily pull out of mud and dung walls. Furthermore, conical ITNs can be tied up during the day to free up interior space needed for daytime activities. Given that limited interior space is also a barrier to use, it may be worthwhile to explore the idea of designing ITNs for outdoor use (e.g., men guarding animals at night).

The qualitative finding that perceived risk of malaria is low in some communities may be due to the pattern of malaria in Ethiopia. In parts of Ethiopia, malaria is epidemic - rather than seasonal or endemic - meaning that every five to eight years, periods of low to moderate transmissions are punctuated by epidemic outbreaks resulting in high morbidity and mortality. The last major epidemic year was in 2003. After prolonged periods without epidemic outbreaks, malaria may recede as a critical problem in the mind of the public. Even when family members get malaria, they can be effectively treated, which may create the perception that malaria is not a serious illness.

Finally, it is worth noting that some reasons for non-use that are found in other countries were not important factors in this study. Extremely few people stated that they did not use ITNs because they were too hot or "suffocating", or that the chemical on a treated net might be dangerous for a pregnant woman or young child.

There are several caveats to this study. First, cross-sectional surveys such as the one presented here can show associations but do not show causal pathways; future studies can be designed to elucidate causal pathways for ITN use and non-use. Second, for programmatic monitoring and evaluation purposes, this study was conducted in areas where substantial ITN promotion efforts had been done to complement free ITN distribution by the FMOH. These areas are likely to have had higher ITN ownership than elsewhere in Amhara or Oromia and in other parts of Ethiopia. Thus, the study cannot be considered typical of ITN ownership and use across the country. Third, some of the factors investigated may be an indicator for poverty in the study population (e.g. malaria knowledge and perceptions) and, hence, may be confounded by poverty and other socio-economic variables that were not sampled. Finally, the number of regression analyses carried out will have increased the odds of finding significant associations; however, only those associations statistically significant in both univariate and multivariate analyses are reported here as having a significant association with use.

## Conclusion

This study demonstrates that in Ethiopia ITN use is associated with a number of factors related to household background, respondent's knowledge, and ITN characteristics, some of which should be incorporated into programme policy. Based on these findings, ITN use in Ethiopia could be increased were conically-shaped ITNs to be distributed. Since the analyses found that ITNs that were paid for were more likely to be used than those obtained free, a segmentation strategy targeting free ITNs to rural and poorest households combined with support for the commercial sector in urban and better-off areas may optimize ITN coverage as well as help increase ITN use. Furthermore, replacement schemes for worn-out ITNs, assistance with hanging ITNs, and communication addressing misperceptions about malaria and ITNs, are likely to raise levels of use.

## Competing interests

The authors declare that they have no competing interests.

## Authors' contributions

CB designed the survey, trained the research team and oversaw the fieldwork, and wrote the original study report that preceded this paper. SW and RR conducted the quantitative data analysis; CB conducted the qualitative analysis. All authors contributed to writing the manuscript, and all read and approved the final manuscript. The opinions expressed are those of the authors and may not reflect the position of their employing organizations or of their funders.
